# Resilient in adversity: Innovative treatment support interventions in facilitating ART adherence among young adults living with HIV in the informal settlements of Kibera, Nairobi

**DOI:** 10.1371/journal.pone.0322823

**Published:** 2026-03-02

**Authors:** Odylia Muhenje, Charles O. Olungah, Dalmas O. Omia, Raphael O. Ondondo, Paul Waswa, Adelaide Lusambili

**Affiliations:** 1 Institute of Anthropology, Gender and African Studies, Faculty of Arts and Special Sciences, University of Nairobi, Nairobi, Kenya; 2 School of Public Health, Biomedical Sciences and Technology, Masinde Muliro University of Science and Technology, Kakamega, Kenya; 3 Strathmore University Business School, Nairobi, Kenya; 4 Environmental Health And Governance Centre, Africa International University, Nairobi, Kenya; Institute of Tropical Medicine / University of Antwerp, BELGIUM

## Abstract

**Background:**

Human Immunodeficiency Virus/Acquired Immunodeficiency Syndrome (HIV/AIDS) poses significant health challenges. Despite ongoing efforts to scale up Antiretroviral therapy (ART) to save lives from AIDS-related morbidity and mortality,adherence among young adults remains low at 65% in sub-Saharan Africa.

**Aim:**

Our study aimed to inform the development of youth-friendly, context-sensitive programs that are both scalable and sustainable in urban informal settlements. This paper presents findings on youth facilitators to ART adherence in Kibera informal settlement.

**Setting:**

The study was conducted in Nairobi urban informal settlement of Kibra, Nairobi county, Kenya.

**Methods:**

We utilized in-depth interviews (n = 25) and key informant interviews (n = 10) to collect data on faciliators to ART adherence among purposively selected eligible partcipants. Thematic analysis framework was employed to analyze the data.

**Results:**

HIV adherence facilitators encompassed patient-related factors like self-acceptance and open disclosure, health system factors such as accessibility, provider relationships, and patient reminders, and socio-economic aspects including support groups, education awareness, family support, and economic empowerment.

**Conclusions:**

The findings emphasize the need for tailored, age-appropriate, and context-specific interventions in informal settlements to enhance HIV adherence. Fostering self-acceptance, creating an enabling environment through youth friendly services, improving provider-patient relationships, and leveraging social support systems such as peer groups and educational initiatives are essential strategies. Integrating these elements into programmatic and policy frameworks will ensure that interventions resonate with the unique realities of this populationpromoting sustainable adherence outcomes.

**Contribution:**

The findings will provide a foundation for developing context-sensitive interventions and programs to improve adherence in similar settings.

## 1. Introduction

According to several studies, HIV/AIDS remains a significant global public health challenge, with sub-Saharan Africa (SSA) bearing the highest disease burden [1,2]. Young adults, particularly those aged 15–24, are disproportionately affected [3]. According to UNAIDS 2024 HIV estimates, nearly 5 million youths were living with HIV in SSA, accounting for approximately 60% of the world’s HIV-positive youth population [4]. In 2023, Kenya’s HIV prevalence was 3.3%, higher among females (4.5%) than males (2.2%). Of 1.38 million people living with HIV, 85% were adults (25+), 10% youth (15–24), and 5% children (<15). New infections totaled 16,752, with 48% in adults, 31% in youth, and 21% in children [5]. This rate of infection among young adults highlights the vulnerabilities among this population of young adults [6]. The global HIV prevalence is 1.2% with the largest prevalence of 9.0% reported in sub-saharan Africa [7].

Despite ongoing efforts, antiretroviral therapy (ART) adherence among adolescents and young adults (AYAs) in sub-Saharan Africa remains alarmingly low at 65% [3,8]. This represents a significant drop from 84% [9]. Despite the fact that retention in care and consistent treatment are essential to achieving UNAIDS’ 2030 targets, young adults in Africa continue to struggle from social stigma and peer pressure to the intense psychological challenges of adolescence that makes it difficult for AYAs to maintain consistent treatment [10–12].

Adherence is the degree of suitability between a patient’s drug taking behaviours, their medical advice and prescriptions which is key to the effectiveness of ART, a crucial component of stabilizing the immune system’s defense towards HIV infections [13]. Research indicates that adherence to ART (antiretroviral therapy) in HIV care are significantly influenced by a complex interplay of individual, social, and structural factors. Key influencers include the patient’s psychological health, supportive HIV services, and the availability of social support from family and community networks [10,14]. Nevertheless, identifying individual factors associated with recent HIV infections may also be useful for targeting HIV prevention among those at risk [15]. However, this research is limited in scope as little has been done in the low middle income slum environments.

To address ART challenges across SSA, this research points out several intervention strategies that have been implemented. Studies have shown that interventions targeting social support systems—such as peer-led initiatives, psychosocial support programs, peer counseling, and community-based approaches—can improve ART adherence among young people by reducing stigma and providing positive role models [16]. Supporting these findings, previous research has demonstrated that technological interventions—such as mobile health (mHealth) applications and phone short message service (SMS) reminders—are effective in promoting ART adherence by offering consistent reminders and enhancing engagement with care services [17].

Several other studies highlight the impact of simple, supportive tools and relationships in helping people stay on track with their ART regimen [18,19]. Small reminders, like setting an alarm, can make a big difference in ensuring doses aren’t missed [10,19]. Sharing HIV status with trusted individuals creates a supportive network that reduces isolation and builds accountability. Trusting, open relationships with healthcare providers who listen and communicate genuinely can also strengthen one’s commitment to care. In support of these findings, other studies show that mHealth programs significantly boost HIV testing, improve ART adherence, and encourage safer sexual practices [20,21]. This study [22] further emphasized the effectiveness of instructional materials and reminder messages as simple yet powerful tools for ART adherence. Together, these studies underscore the importance of both human connections and accessible tools in enhancing HIV care.

In Kenya, recent reports indicate that youth contributed to the 31% of new infections within Nairobi county [5]. Kibera slums has an estimated HIV prevalence of 12% [23] and is characterized by poverty, lack of basic services such as water and housing, social exclusion and economic deprivation [24].

Kibera, one of the largest informal settlements in Africa, presents distinct barriers to healthcare due to pervasive poverty, limited access to medical services, and overcrowded living conditions [25]. These challenges create significant obstacles for young adults living with HIV, making it particularly difficult to maintain consistent adherence to antiretroviral therapy (ART). While evidence suggests that environmental stressors in Kibera increase the likelihood of treatment interruption and intensify stigma and social isolation [26] the context-specific insights into the facilitators of adherence remain limited. To address this gap, we conducted ethnographic research to assess the facilitating factors influencing ART adherence among young adults in Kibera. This paper focuses specifically on the facilitators of ART adherence with the aim to inform the development of youth-friendly, context-sensitive programs that are both scalable and sustainable in urban informal settlements, to improve health outcomes for young adults living with HIV in Kibera and similar communities.

## 2. Materials and methods

### Study design

This study used a qualitative design to investigate the facilitators that impact ART adherence among young adults aged 18–24 years residing in Kibera informal settlement in Nairobi, Kenya. The combination of both key informant interviews (KIIs) and in-depth interviews (IDIs) proved valuable for capturing the complex factors shaping young adults’s health-seeking behavior concerning ART, especially in the context of ongoing stigma and discrimination faced by this population..

### Study setting

The study was done in Kibera informal settlement, located in Kibra sub-County, Nairobi City County, approximately 5 kilometers southwest of Nairobi central business district. Kibra consists of five wards: Sarangombe, Woodley/Kenyatta Golf Course, Makina, Laini Saba, and Lindi. With over 185,777 residents and a population density of 15,311 people per square kilometer, Kibera is one of Africa’s largest informal settlements, covering 12.1 square kilometers [27]. The area is characterized by overcrowding, informal housing (made from mud and corrugated iron), limited access to sanitation and clean water [25] and high levels of unemployment, with many residents depending on unstable informal economies. These challenging socio-economic conditions, combined with high-risk behaviors, contribute to the high prevalence of illnesses, including HIV and AIDS [6].

The Kibera Community health centre (KCHC), a level 3 health facility established with support from the African Medical Health Research Foundation, plays a vital role in providing healthcare services in this context. As the largest health facility in the area, KCHC serves all the villages in Kibra and was the first comprehensive facility in Kenya to offer antiretroviral therapy (ART), starting in 2003. The center addresses a range of health challenges, including tuberculosis and HIV, amidst the significant socio-economic struggles faced by the community in this informal settlement. KCHC’s role in managing health issues, particularly HIV, offers insights that could inform programming for young adults living with HIV in other similar contexts across Kenya and Africa.

### Study population and sampling strategy

The process began with the selection of a primary health facility. KCHC was selected for its role as the main provider of HIV and tuberculosis care in Kibera. As the first facility in Kenya to offer antiretroviral therapy (ART) since 2003, it provides key insights for supporting young adults living with HIV in similar contexts. Eligible participants were identified through a linelist from the facility Electronic Medical Records (EMR), approached for study participation during their normal clinic visits and a face-to-face study interview scheduled for those willing to volunter. The inclusion and exclusion criteria for young adults living with HIV (YPLHIV) has been highlighted in Table 1 and in Table 2.

**Table 1:** Inclusion and exclusion criteria YPLHIV.

CRITERIA	INCLUSION DETAILS	EXCLUSION
**Age group**	Young adults aged 18–24	Below 18 and above 25
**Duration of HIV treatment**	Receiving HIV treatment in Kibra for at least 12 months before the study	Receiving HIV treatment for less than 12 years. Not residing in Kibera
**Treatment history**	History of treatment defaulting but traced back on treatment, or consistently on treatment with no defaulting	

**Table 2:** Inclusion and exclusion criteria for HCWs in the study.

Inclusion criteria	Details
**Health care providers experience**	At least six months of experience providing HIV care services within Kibra

Focusing on individuals aged 18–24 years captures a demographic population that is transitioning from adolescence to adulthood, a phase marked by unique social, emotional, and economic challenges that may influence their health-seeking behavior and ART adherence. By selecting participants with at least 12 months on HIV treatment, the study encompasses individuals who have encountered various stages of the ART journey, which include being tested for HIV, linked to care, initiated on antiretroviral therapy (ART), and adhering to treatment, all of which are crucial for achieving viral suppression and ensuring adherence to care. This offers insights into the long-term factors that support adherence. Including both those with a history of treatment defaulting and those with consistent adherence enables the study to explore contrasting experiences between stable (adherent and active with no treatment interruption) and unstable (with episodes of interruption of ART treatment).

From electronic medical records (EMR) a total of 148 young adults were identified that were active in the hospital EMR. Of these 132 young adults had been on treatment for the last 12 months. The distribution of the 132 was 57 individuals with a history of defaulting and 75 without a defaulting history were identified, with a gender breakdown of 19 males and 38 females in the defaulting group, and 32 males and 43 females in the non-defaulting group. We then finally purposively selected 25 participants distributed as 4 male, 7 female, 6 male and 8 female who were sampled using probability proportional to size from the 19 male, 38 female, 32 male and 43 female respectively.

### Consenting process

Before initiating KIIs and IDIs, the research team engaged participants on the study’s aims. This initial interaction aimed to foster transparency ensuring participants had a clear understanding of the research purpose and their role within it. Participants were presented with an informed consent form, which was either read aloud or provided as a written document. One male participant with epileptic condition was read to and a total of 34 participants read on their own. These forms meticulously outlined the potential risks and benefits of participation, ensuring alignment with ethical research standards. Written consent was obtained from all participants. As an ethical protocol, the interviewer also signed the consent form and provided a copy to the participant for their personal records.

To accommodate linguistic diversity, the consent forms and interview guides were offered in both English and Kiswahili. In cases where participants had questions or concerns, researchers addressed them comprehensively prior to seeking consent. The interviews were conducted in private settings within health facilities, selected to ensure privacy and safeguard the comfort of participants. Each session lasted approximately one hour,

### Data collection

Study recruitment for this study was conducted from 6^th^ September 2022 to 20^th^ January 2023, and data collected employing multiple qualitative methods, including in-depth interviews (IDIs) and key informant interviews (KIIs) at the clinic that lasted for 45–60 minutes. The qualitative tools were initially developed in English and subsequently translated into Kiswahili to cover different demographic populations. Before formal data collection commenced, the research team conducted a pilot study between 28^th^ July 2022 and 16^th^ August 2022, to validate and test the effectiveness and clarity of the qualitative tools. This piloting process involved using the tools with a small group of participants to identify any issues or ambiguities in question phrasing, structure, or flow. Feedback from the pilot highlighted the need to refine certain questions to be more culturally relevant and less ambiguous. For instance, questions related to ART adherence required clearer language around medical terms to ensure participants fully understood them. Additionally, the pilot revealed the need for more prompts to facilitate open discussions about sensitive topics, such as stigma, sexual and gender based violence (SGBV) and family dynamics. Based on these insights, the tools were revised to improve clarity and better capture the study’s objectives.

The research team underwent a three-day training session led by the principal investigator (PI), who is an experienced social behavioral scientist. The training covered essential qualitative interviewing skills, including role-playing exercises to simulate real interview scenarios. These exercises allowed interviewers to practice and refine their questioning techniques, particularly focusing on maintaining neutrality and active listening. The training also emphasized probing skills, teaching interviewers how to ask follow-up questions that encourage participants to elaborate on their responses without leading or influencing them. Sensitivity training was also a critical component, guiding interviewers on how to handle delicate topics like stigma, mental health, SGBV and family support with empathy and respect. By incorporating these elements, role-playing, probing techniques, and sensitivity training,the team was well-prepared to conduct interviews that were both comprehensive and respectful of participants’ experiences and perspectives.

Conducted in Kiswahili at the Kibra Community health facility, each IDI lasted approximately one hour. These interviews focused on barriers to ART linkage, adherence, retention, and other related issues, including stigma, misconceptions, and support systems. Key informant interviews were conducted by OM in English with clinical healthcare providers (HCPs) and in Kiswahili for non-clinical HCPs. These interviews explored similar themes to gain insights from healthcare professionals involved in HIV care. With participant consent, all interviews were audio-recorded, and the interviewer took notes to capture additional context and non-verbal cues. Interviews were held in private settings within the health facility. Confidentiality was prioritized by conducting the sessions in secluded areas to protect participants’ privacy.

### Data analysis and management

The recorded audio files were transcribed verbatim. The accuracy of the transcripts was validated by OM randomly comparing them with the original audio files. All data were securely stored on password-protected laptops. The data were then imported into NVIVO [Version-14] for analysis. The data analysis process utilized a deductive thematic analysis framework based on the World Health Organization (WHO) dimensions of adherence. This structured approach focused on organizing and coding data according to established factors related to patients, healthcare systems, socio-economic conditions, therapy-related issues, and disease characteristics. Emerging sub-themes were systematically categorized within these dimensions, ensuring a comprehensive understanding of ART adherence among participants.

The analysis began with familiarization, where OM and WP independently reviewed initial transcripts to immerse themselves in the content. These codes were then grouped into broad categories, which were iteratively reviewed and refined to develop a robust coding tree. Emerging sub-themes were organized into three key groups; patient-related factors, health system factors, and socio-economic factors, all of which were identified as facilitators to ART adherence. The study team concurrently conducted open coding to capture emerging themes drawn from participants’ narratives during in-depth interviews and key informant interviews with the young adults living with HIV. This dual approach enabled the study team to organize the data into a hierarchical structure of the three main themes of facilitators. OM and WP, serving as independent coders, conducted the coding process separately and reached consensus on the final thematic and sub-thematic areas through frequent discussions and comparison, ensuring rigor and intercoder reliability. The final themes were defined, named, and prepared for the last stage of analysis, culminating in a comprehensive examination of ART adherence facilitators. The systematic use of both deductive methods, along with the structured Braun and Clarke’s six-step analysis process, allowed the study to reach data saturation, ensuring a robust and in-depth exploration of the factors supporting ART adherence.

### Ethical approval

Before data collection began, the study was approved by the ethical review committee (ERC) of Kenyatta National Hospital and the University of Nairobi (reference number P/500/06/2021). Additional permission was granted by the National Commission for Science, Technology, and Innovation (NACOSTI) under license number NACOSTI/P/22/19231. The study further received ethical clearance from the Nairobi Metropolitan Services – Health Directorate’s research ethics committee (REC) under clearance certificate number EOP/NMS/HS/199, and Kibra Sub-county, with approval from the facility in-charge.

## 3. Results

### Characteristics of participants

A total of 35 interviews were conducted which included 25 IDIs with young adults living with HIV (10 male and 15 female) and 10 KIIs with 10 HCPs, (5 clinical and 5 non-clinical) young adults as demonstrated in Table 3 and in Table 4 outlining the socio-demographic characteristics of 25 young participants in the study, with a mean age of 22 years. The majority of participants (56%) were aged between 21 and 24 years, and females represented 60% of the sample. A significant portion (72%) resided with their parents, while 88% identified as single. Regarding educational attainment, 64% had completed secondary education, and 56% reported living with HIV for a duration of 0–10 years. Additionally, 52% had been on antiretroviral therapy (ART) for 0–10 years, with 56% having no history of treatment default. These demographic insights are crucial for understanding the lived experiences of young individuals on ART by providing context.

**Table 3:** Socio-demographics for the IDI participants.

Participant characteristic	Range	Frequency	Average	Proportion (%)
**Young adults**				N = 25
**Age**	18–20	11	Mean age of 22 years	44
	21–24	14		56
**Gender**	Male	10		40
	Female	15		60
**Residing with**	Living alone	7		28
	Living with Parents	18		72
**Marital status**	Married	3		12
	Single	22		88
**Level of education**	Primary	4		16
	Secondary	16		64
	Tertiary	5		20
**Years since diagnosis with HIV**	0–10	14	9.2 years	56
	11–20	11		44
**Years on ART treatment**	0–10	13		52
	11–20	12		48
**Default history**	Ever defaulted	11		44
	Never defaulted	14		56

**Table 4:** Socio-demographic table for the KIIs participants.

PARTICIPANT CHARACTERISTICS	RANGE	FREQUENCY	PROPORTION (%)
**CLINICAL HCPS**	Clinicians	2	20
	Counsellor	2	20
	Nurse	1	10
**Non clinical HCPs**	MIPA–Adult	3	30
	MIPA–OTZ	2	20
**Gender**	Male	5	50
	Female	5	50
**Age**	20–25	2	20
	26–30	2	20
	31–35	1	10
	35–40	2	20
	Above 40	3	30
**Marital status**	Single	1	10
	Married	6	60
	Separated	2	20
	Widowed	1	10
**Years of service**	0–5	1	10
	6–10	3	30
	11–15	4	40
	16–20	2	20
**Area of residence**	Within Kibra	5	50
	Outside Kibra	5	50

### Study themes

Our findings show a clear hierarchy of factors shaping ART uptake and adherence among young people (Table 5). The most mentioned facilitators were strong provider–patient relationships, support groups (physical and WhatsApp-based), and other game-changing elements, including reminder tools such as alarms, peer education, MIPA, and U = U literacy. These factors were consistent across participants, regardless of prior treatment default, reflecting shared experiences of what supports adherence.

**Table 5:** Reported factors influencing intervention uptake among young people and clinical/non-clinical providers (n = 35).

Ranked Interventions (Most to Least Mentioned)			
Rank	Theme/ Sub-theme	Number (n = 35)	Percentage (%)
1	Provider–patient relationship	26	74
2	Support groups (physical or WhatsApp)	24	69
3	Patient reminders (SMS, alarms, cards)	23	66
4	Acceptance	22	63
5	Counseling	21	60
6	Accessibility (proximity, convenience)	20	57
7	WhatsApp/technology-based support	19	54
8	Open disclosure	18	51
9	Peer education & awareness	18	51
10	U = U literacy	17	49
11	Family support	16	46
12	Availability (injectables, 3-month refills)	15	43
13	MIPA (Meaningful involvement of people living with HIV)	14	40
14	Economic empowerment	12	34

Participants also highlighted self-acceptance, open disclosure, proximity to clinics, reliable medication access, and practical reminders (e.g., alarms or texts) as important for daily adherence. Peer support, especially via WhatsApp, was described as a key motivator, offering guidance, affirmation, and a sense of shared experience.

Notably, U = U literacy emerged as a novel science and game-changing insight. Although few had encountered it before, participants described it as the single most important factor motivating testing, early linkage, and sustained ART adherence, reducing stigma and fostering confidence. Least mentioned factors, such as broader structural supports, were valued but secondary to the relational, peer, and literacy-based anchors central to young people’s lived experience of adherence.

### Patient related factors

#### Acceptance.

Our findings showed that patient-related factors such as “acceptance’ and “open disclosure” influenced their adherence to ART. The role of self-acceptance, which empowered individuals to take ownership of their health and commit to their treatment, was key. For many young adults, consistent access to self-care resources, counselling, and basic needs like food can foster self-love and resilience, as one young respondent expressed.

*“…one has to go for counselling, so that you can take care of yourself, you love yourself, self-acceptance of your status,then it will not be difficult to take those drugs, you just have to remember its now time to take drugs and you do it.”(IDI_YWLHIV)*

Similar views were also echoed from the KII interviews:

#### Open disclosure.

Disclosure emerged as a critical facilitator, as mentioned by most respondents, particularly in the context of developing trust and social support networks. Healthcare workers highlighted challenges in coordinating care for children within the school system, often arising from parents not informing teachers in advance. Facilitating adherence can be achieved by ensuring that teachers are informed beforehand, which enables them to support children in taking their medications or accessing healthcare services.

As one healthcare provider noted:

*“Some parents fear that they have not discussed the HIV status of their children with the school administration. Even if the child is denied permission to leave, sometimes the teachers are not even aware of the situation. However, I know a few school administrators who will allow you to pick the child up for an appointment or bring the medications. I will speak to those school administrators about this issue to encourage better communication and understanding between parents and teachers. However, if you have not discussed the situation with the teacher, you cannot blame the teacher”* (KII_HCP_).

The young adults in the study expressed views that aligned with those of the healthcare providers, noting that effective communication with teachers is crucial for facilitating timely access to healthcare within school schedules. One of the young participants noted;

*“Sometimes, teachers are working with a very tight schedule, especially during periods when they are conducting Continuous Assessment Tests (CATs) or preparing for end-of-term exams. In such cases, they may deny permission and tell you, ‘Not today; come back next week,’ because this week is reserved for exams. However, if you explain the situation regarding your child, they may advise you (parent) to come and pick them(child) up this week on Friday, as there will be no exams then. This allows you to bring the child in for an earlier appointment before the exams. Essentially, it all depends on effective communication”* (IDI_YFLHIV).

Additionally, the importance of educating young adults living with HIV about their treatment was emphasized by another respondent. They stated,

“*You are supposed to inform them [young adults] about the medications. They need to understand that their lives depend on these drugs, and they should adhere to their treatment”* (IDI_YLWHIV).

This highlights the necessity of ensuring that young adults are aware of the significance of medication adherence for their health outcomes.

Many young adults emphasized that seeing and hearing about others living with HIV, peers who have successfully navigated similar challenges served as a powerful motivator. Role models of those who have remained consistent with their treatment and maintained good health provide tangible proof that a fulfilling and healthy life with HIV is achievable. Participants shared that success stories of individuals who have thrived while adhering to their treatment can serve as powerful motivators. One respondent remarked,

*“There are success stories that people have encountered in life while still taking the same medication. You should take a moment to put yourself in their shoes;they feel if I continue doing this, I will be healthy” (IDI_YWLHIV).*

Another participant emphasized the importance of empowerment and positive reinforcement for those newly diagnosed with HIV. They stated,

*“What will truly make them happy is empowering them by informing them that there is a fulfilling life after being tested HIV positive. By providing examples and sharing the positive experiences of individuals who have prospered, I believe that will bring them hope and happiness” (KII_HCPp_).”*

### Health system factors

#### Accessibility.

Our findings suggested that access to nearby healthcare facilities may support ART adherence among young adults living with HIV. Proximity to a clinic not only facilitated easy access to medication and information but also provideda sense of reassurance when health concerns arose. As one participant shared:

*“definitely even as I had stated earlier, if these youths can maybe access these services at the door step and feel like, I do not need to walk/strain an extra mile to access these services, I will be comfortable to have them. Imagine you are HIV positive and you are now told, you do not need to come to the facility, we will serve you at your comfort where you are in your house, we will do viral load there, we will measure your weight, pressure, drugs at your house yet you are not bedridden, you can do anything just as any other person, but you are just being treated in a special way”* (IDI_YMLHIV).

*“Proximity to the clinic contributes in that if you are near the clinic and you have an issue, you easily go to the clinic and get information”* (IDI_YMLHIV).

As shown in the above quote, some young adults still chose to travel to distant clinics due to stigma, fear of being identified, and a desire to keep their HIV status private. These sentiments underscored the need for accessible healthcare that also respected young adults’s privacy and provided a non-judgmental environment, encouraging them to stay engaged in their care.

Being part of a supportive community where they can share experiences and receive encouragement was invaluable. As one participant expressed

*“If I can get that [Support groups], I can be very happy because there are youths here... you encourage them and tell them how those drugs work.”* (IDI_YLWHIV).

*“The support groups provide a sense of community and solidarity, reminding individuals that they are not alone in their journey.”* (KII_HCP Clinical_F).

#### Availability.

A significant number of young adults noted that injectable treatments would be more convenient and preferable compared to the daily pill regimen and that this would make adherence easier and more manageable,

*“I think they would also prefer that(Injectables).. So, I think if we were to get to that level of having the injection, then we will embrace it... it will be better compared to the daily taken pill”* (IDI_YWLHIV).

In addition commodity distribution models where the young adults were given drugs that last upto 90 days was reported to encourage adherence as they would not be required to visit the facility every now and then, as reported by one of the young adults:

*“I feel that the approach where you are given (ARV mdedicines) for three months is good because even if one plans to travel somewhere, you don’t have worries that I will run out of drugs while away and for those three months, it will stick in your mind that you have an upcoming appointment somewhere.” (*IDI_YWLHIV*).*

#### Healthcare provider-patient relationship.

Positive provider-patient relationship was reported to promote adherence. Healthcare providers reported the importance of interacting with the patients based on their level to ensure tailor-made information as well as build a positive interaction, understanding their issues and the challenges they are going through.

*“Understanding the client means sometimes this client may come from outside the catchment area. So, if they tell you that this time, please don’t give me drugs for one month, just give me* *two months or three months, please understand, he might be saying the truth and number two if they tell you that please can I just have those drugs from your office because I don’t want to go to the pharmacy, please understand” (KII*_HCPp_).

*“So it depends on how you interact with them and the rapport you make with them. If you interact with them at their level, you understand their issues and challenges and then you counsel them in a friendly way, then they are more likely to accept the linkage as opposed to when they (health providers) are a bit rough or rigid”* (KII_HCPc_).

In addition, some of the respondents also cited being comfortable interacting with healthcare providers of their age than those who are older than them.. This was reported to boost disclosure as well as recounted by one clinical healthcare provider:

*“Maybe the healthcare providers should be of the same age range... as they fully understand each other.”* (KII_HCPc)

Counseling emerged among many of the respondents as a critical intervention for young adults living with HIV, transforming their perception of the virus from a life-ending diagnosis to a manageable condition. By providing personalized support, emotional reassurance, and community connection, dedicated counselors helped build trust, enhance treatment adherence, and address the complex psychological challenges associated with HIV. One respondent noted in a KII,

*“You need to have more counsellors so that people are allocated their own counsellors... that will really encourage the youth.”* (KII_HCP_nc)

*“The one talking to me made me so happy because he called in two other counsellors who talked to me and they told me that it is not the end of life if I adhere, I will live a long life and I should always know that I am not alone,and that made me very happy.* (IDI_YWLHIV).

#### Meaningful involvement of Young adults living with HIV/AIDS (MIPA) and Undetectable = Untransmittable (U=U) literacy.

MIPA in their health and Youth friendly cornerswas acknowledged by respondents as playing an important role of health interventions in promoting adherence to ART. As one clinical provider shared:

*“Engaging YPLHIV in decision-making about their own lives and the lives of people in their communities empowers them and enhances their knowledge and skills on matters related to HIV and AIDS.”* (KII_HCP_c)

*That’s where (MIPA activitivies) they share more, they’re empowered. In fact, they’ve given information of people living with HIV and how to take care of themselves, what to do, and even in fact on general adherence.* (KII_HCPc_F_)

“U=U” (Undetectable = Untransmittable) literacy emerged as a pivotal theme among majority of the participants, highlighting its transformative impact on adherence to ART and perceptions of living with HIV. For many, while the message of U = U was novel and not well understood by most young people, it was a gamechanger that empowered them to regain control over their lives and health,enabling them to combat stigma and live positively, fostering a sense of autonomy and optimism. A participant shared,

*“U=U is not a cure for HIV, but that if I have suppressed the virus to a point where it doesn’t have power over me. I am the one controlling it.”* (IDI_YLWHIV)

Healthcare providers emphasized the practical implications of U = U, particularly in promoting safe childbearing and reducing transmission risks in intimate relationships. One healthcare provider noted,

*“They (young people) know that once they are at U=U, they will have a chance to have unprotected sex with their partner and reproduce an HIV-free and healthy baby.” (*KII_HCP_)

Moreover, the integration of U = U education into community outreach and clinic messaging has significantly contributed to normalizing this important concept. One young participant shared their journey, recounting their initial skepticism about the U = U message but later reflected, on how their undersdaning evolved, recognizing he profound implications of U = U for both personal health and the broader community. This shift in perspective underscores the effectiveness of educational efforts in fostering acceptance and promoting informed discussions around HIV prevention and treatment. As one young respondent shared her experience;

*“When I finished school, I started doing my research on it(U=U), and that is when I realized it was working for me.”* (IDI_YLWHIV)

#### Patient reminders.

Patient Reminders emerged among majority of respondents as a crucial theme, with various methods highlighted for ensuring adherence. Appointment cards, SMS reminders, and calendar alarms were commonly used tools, enabling patients to keep track of clinic visits and medication schedules. One participant noted:

*“I use the alarm on my phone to remind me to take my medication before leaving for school. It helps me stick to my schedule.”* (IDI_YLWHIV)

Another clinical healthcare provider remarked that:

*“We usually have appointment cards to remind them of their clinics, they also mark on their calendars, and we also sent them SMS to remind them of their clinics.” (*KII_HCPc_)

Implementation of patient reminders enhanced clinical shedules for young people as noted by one respondent;

*“she was registered even for clinical reminder, o that incase she forgets her clinical day she will just gate the message on her phone that she is supposed to come for her treatment”* (structured clinic observation_)

Messages and alarms were widely accepted, with participants appreciating their utility. One respondent mentioned the role of text messages for adherence (T4A) in managing busy schedules:

*“Text messaging is effective in reminding YPLHIV about their clinic appointments, as many young adults are constantly on their phones.”* (IDI_YLWHIV).

*It’s also nice to be reminded because may be people have too busy schedules, so to be reminded is a good thing……….I think that’s a way of reminding and it’s really nice because even if you had forgotten or even had not but then someone texts, you will feel this person cares.* (IDI_YLWHIV).

Majority of the youth also reported that they have been leveraging on technology, such as through WhatsApp groups to access information, ask questions so as to get answers for things that bothered and affected them. They reported that the platform allowed for easy access of information and quick responses. This is described by one young male living with HIV:

*“WhatsApp groups offer a convenient platform for YPLHIV to connect, share information, and provide mutual support, thereby reinforcing adherence to ART.”* (IDI_YMLWHIV).

### Socio-economic factors

#### Support groups.

Sustainable support was identified as a pivotal factor in promoting adherence to antiretroviral therapy (ART) among young people living with HIV (YPLHIV). The majority of participants reported several key aspects of the support they require, highlighting their significance in facilitating consistent adherence to treatment. These aspects included emotional support, access to information, and assistance with navigating healthcare systems. The findings underscore the diverse needs of YPLHIV and emphasize the importance of a comprehensive support system in enhancing their health.

Support groups were reported to create an environment conducive for meaningful engagement and peer interaction. As expressed by a female young person in an IDI:

*“I’ve been to physical support groups and I have been to a WhatsApp group for a support group. And it’s very nice because even if you are losing faith, you will get time to ask someone a question that has been bothering you and because in that support group you find a team of your age and all of you express different problems and at times you find yours is better, and you express yourself and you get the answer for your problems. So, you get answers and you know that I shouldn’t be doing this to avoid this, I should do this so that this happens. So at the support group you have your friends to give you morale while backing you up to do this and that which is nice.”. (IDI_YFLHIV).*

*“Support groups provide a platform for YPLHIV to connect with peers, share experiences, and learn from one another, ultimately encouraging adherence to ART.”* (KII_HCPc_).

*“The support groups provide a sense of community and solidarity, reminding individuals that they are not alone in their journey.”* (KII_HCPc_).

*“in their peer gatherings, they do share the good side stories, okay, for those that have got information that may be one is living positively, and then physically seeing the person is growing well, having that good health always encourages them”* (KII_HCPc_).

Peer support groups for young adults living with HIV, such as Operation Triple Zero (OTZ), were seen by majority of respondents as vital, providing not only a sense of community but also practical reminders about adherence and the importance of keeping appointments. As one participant explained,

*“Yes, OTZ talks about zero missed appointments, zero missed drugs, so it leads to viral suppression.”* (KII_HCPc_).

*Yes, OTZ is Operation Triple Zero, it is usually a group of young adults living with HIV where they are talked to about zero missed appointment, zero viral load, zero misseddrugs…….It is usually like a support group, young adults interact and do activities together, they share challenges and it is like a safe community for these young persons living with HIV.* (KII_HCPc_).

#### Peer education and awareness.

Education and awareness were consistently noted as crucial interventions, particularly through healthcare champions. Peer educators and peer mentors acted as role models, sharing their personal journeys and helping young adults understand the importance of medication adherence. As one respondent explained,

*“Education empowers YPLHIV to understand the importance of adherence and encourages them to remain engaged in HIV care services.”(*IDI_YMLWHIV)

*“Peer educators and champions serve as role models, sharing their personal journeys and providing encouragement to YPLHIV, thereby fostering adherence to ART.”* (KII_HCPc_)

Another young female living with HIV shared the following on education and mentorship:

*“The first thing I always say is that it(peer education) will educate the youths…it is important that we adhere to medication so that we can be well”.* (IDI-YFLWHIV)

*“Linkage to some of the treatment on a literacy treatment training will encourage then getting more empowered by the peer educators.”* (KII_HCPc_).

#### Family support.

Family support, both financial and emotional, was another critical enabler. Participants expressed how family support reassured and motivated them to adhere to their treatment. One participant noted,

*“You can be reminded by your spouse because phone batteries die, we had something like a tin that had dates and that is where we put our phones wallet, so once you pick those things (ARVs) that you use daily, it reminds you of the date and you need to take your drugs with you to take at a certain time.” (IDI- YPLHIV)*

#### Economic empowerment.

Providing economic empowerment opportunities for YPLHIV contributes to their overall well-being and enhances adherence to ART. By supporting income-generating activities within established support groups, individuals gain financial independence, enabling them to afford transportation to healthcare facilities for ART refills and appointments. As non-clinical healthcare provider (Peer educator) in a key informant interview highlighted,

*“Empowering YPLHIV through income-generating activities not only fosters economic stability but also promotes a sense of self-reliance and motivation to adhere to treatment.” (KII*_HCPp_).

*“Because you go to these organizations that I talked about, they usually do an assessment then they motivate you either by giving you food baskets or they decide to open a business for you” (KII*_HCPp_).

## 4. Discussion

This study provided a nuanced exploration of factors influencing ART adherence among (YPLHIV), underscoring the intersection of patient, health system and socio-economic factors that shape ART adherence behavior.This research highlighted how adherence is more than a medical routine.

### Acceptance and open disclosure

Acceptance of one’s HIV status emerged as a critical first step toward adherence. YPLHIV who achieved self-acceptance tended to adhere to ART because they viewed treatment as a pathway to maintaining a healthy, fulfilling life. This process of acceptance was often supported by open disclosure, where young adults shared their HIV status with trusted individuals such as family members, peers, or healthcare providers. Disclosure reduced feelings of isolation and stigma, fostering an environment where adherence could thrive. These findings echo previous anthropological work suggesting that disclosure is not merely an individual decision but a social one shaped by clinical and demographic characteristics [28]. In this study, the ability to disclose without fear of discrimination was key to accessing social support systems that reinforce adherence. In addition, these findings were similar to studies in SSA on adherence [29,30].

### Role of health facility factors and patient reminders

Participants described various reminder systems, including appointment cards, text messages, and alarms, which helped them stay on track with medication schedules. These methods aligned with structural factors within the health facility that further enabled adherence, such as proximity to care, flexible appointment scheduling, and the availability of youth-friendly services. The findings point to the importance of creating health systems that are responsive to the unique needs of YPLHIV. Healthcare providers played a crucial role in supporting adherence by offering consistent, youth-centered care and ensuring that medication was readily available and accessible. Regular reminders through various channels such as alarms, phone calls, calendars and mobile short text message reminders [31] are effective in improving adherence and retention among people living with HIV [32,33].

### Impact of healthcare worker attitudes and patient-provider relationships

The attitudes of healthcare workers and the nature of patient-provider relationships significantly influenced adherence behaviors. Participants who described positive, empathetic interactions with their healthcare providers were more likely to adhere to their treatment regimens. Healthcare workers who engaged with YPLHIV in a respectful and understanding manner helped reduce the fear and stigma associated with HIV, thereby reinforcing adherence. The role of success stories shared by healthcare workers and peers living with HIV was particularly motivating for YPLHIV, illustrating how narrative and lived experience can be powerful tools for health promotion and behavioral change. These findings were similar to other studies that reported supportive patient to provider relationship was significant as it created trust between the patient and the provider thus fostering free information sharing [29,31].

### The role of social support systems

The role of social support systems emerged as a fundamental theme in fostering ART adherence. Peer-to-peer support groups, such as Operation Triple Zero (OTZ) were cited to offer YPLHIV a sense of community, where they could share experiences, receive encouragement, and witness the success of others on similar treatment journeys. This peer engagement not only provided emotional support but also created an accountability structure that motivated participants to adhere to their medication. These peer groups allowed YPLHIV to navigate their HIV status in a socially supportive environment, mitigating the feelings of alienation often associated with the disease. In addition, shorter turnaround time at the heath facilities [30] with youth friendly services fostered adherence. The results of this study indicate that young adults with sufficient knowledge about HIV testing, linkage and ART adherence including Undetectable equals Untransmittable (U = U) literacy tended to adhere to ART and to be retained in HIV care services. These findings are similar to those from a systematic review citing associations between U = U awareness with good health outcomes among PLHIV in 25 countries [34]. Moreover, this study noted that psychological support provided by family members, healthcare workers, or community members facilitated adherence to ART services, as demonstrated elsewhere [35].

### Peer mentorship, support groups, and family support

Beyond peer support, peer mentorship played a transformative role in ART adherence. Peer mentors, often YPLWH themselves, served as role models and educators, offering tangible examples of successful adherence and viral suppression. This mentorship extended beyond emotional support to include practical advice on managing treatment regimens, thereby fostering a culture of adherence within the YPLHIV community. Similarly, family support both emotional and financial was identified as crucial for sustaining adherence. Families that provided unconditional support, without stigma or judgment, empowered YPLHIV to stay committed to their treatment.Similar findings on importance of peer support have been found in a study in a qualitative study in Western Uganda in which social networks of young adults [18–24] were described to have a great role in combating their perceived barriers to ART adherence [36]. This study highlighted the importance of Meaningful involvement of People living with HIV (MIPA) in supporting couples of YPLWH adhere to ART treatment and be meaningfully engaged. A Netherlands study [37] has similarly highlighted the importance of MIPA in cure research, emphasizing the need for increased awareness, funding, standardized guidelines to ensure meaningful involvement to combat tokenism and misconceptions. Psychosocial and family support, as well as support groups and treatment literacy emerged as key in enhancing ART adherence among the YPLHIV in these settings. These findings are consistent with other studies among YPLHIV in Western Uganda [38].

### The role of economic empowerment and food security

The study highlighted the role of economic empowerment and food security in enhancing ART adherence among young adults living with HIV (YPLHIV) in informal urban settlements. Economic empowerment enabled financial independence, allowing YPLHIV to afford transportation for healthcare visits, thus supporting consistent ART adherence. Engaging in income-generating activities within support groups not only addressed logistical barriers but also boosted psychological well-being, fostering self-reliance and motivation [39]. However, challenges remained in ensuring consistent employment opportunities in informal settlements, underscoring the need for scalable, sustainable programs. In addition, studies showed that incentives such as provision of food and nutritional supplementation promoted adherence [40]. This is consistent with our findings. Furthermore, this research added to the growing consensus on the importance of integrating socio-economic interventions within HIV care programs supporting a holistic approach to ART adherence beyond clinical interventions [41].

## 5. Conclusion

This study illustrated that ART adherence among YPLHIV is shaped by a complex interplay of social, psychological, and structural factors. Acceptance, disclosure, peer support, support groups, U = U literacy and positive healthcare interactions form the bedrock of adherence behaviors, while structural interventions such as patient reminders and youth-friendly health services provide the necessary scaffolding. From an anthropological perspective, adherence is not merely a medical issue but a deeply relational one, embedded within the social worlds of young adults. As such, interventions aimed at improving ART adherence should prioritize MIPA, the development of strong peer networks, supportive family environments, and empathetic healthcare systems that resonate with the lived experiences of YPLHIV.

### Study limitations and strengths

This study is one of the few studies that has been conducted in the urban informal settlements, on youth ART adherence (vulnerable group) and raises key issues on policy implications.

We recognize the limitation of not calculating a Kappa statistic to measure inter-coder agreement. However, we ensured a thorough codebook development, extensive coder training, and implemented a process of resolving any disagreements by maintaining ongoing discussions and consensus building about the results, regular checking of coding discrepancies,codes, and emerging themes to maintain high levels of consistency among the coders and uphold the credibility of the data.

## Supporting information

S1 File COREq checklist 2. (DOCX)

## Abbreviations

HIV: Human Immunodeficiency Virus
AIDS: Acquired Immunodeficiency Syndrome
YPLHIV: Young adults Living with HIV
ART: Antiretroviral Therapy
ARV: Antiretroviral
SSA: Sub-Saharan Africa
UNAIDS: United Nations Programme on HIV/Aids
KII: Key Informant Interviews
HCP: Health-care Provider
IDI: In-depth Interviews
WHO: World Health Organization
SMS: Short message service
U=U: Undetectable equals Untransmittable
MIPA: Meaningful Involvement of People Living with HIV
OTZ: Operation Tripple Zero
T4A: Text for Adherence
